# Who continued travelling by public transport during COVID-19? Socioeconomic factors explaining travel behaviour in Stockholm 2020 based on smart card data

**DOI:** 10.1186/s12544-021-00488-0

**Published:** 2021-06-07

**Authors:** Erik Almlöf, Isak Rubensson, Matej Cebecauer, Erik Jenelius

**Affiliations:** 1 Integrated Transport Research Lab, KTH Royal Institute of Technology, Drottning Kristinas Väg 40, 114 28 Stockholm, Sweden; 2 Trafikförvaltningen (Public Transport Administration), Region Stockholm, Stockholm, Sweden; 3 Division of Transport Planning, KTH Royal Institute of Technology, Stockholm, Sweden

**Keywords:** COVID-19, Public transport, Socioeconomic factors, Smartcard data

## Abstract

**Introduction:**

The COVID-19 pandemic has changed travel behaviour and reduced the use of public transport throughout the world, but the reduction has not been uniform. In this study we analyse the propensity to stop travelling by public transport during COVID-19 for the holders of 1.8 million smart cards in Stockholm, Sweden, for the spring and autumn of 2020. We suggest two binomial logit models for explaining the change in travel pattern, linking socioeconomic data per area and travel data with the probability to stop travelling.

**Modelled variables:**

The first model investigates the impact of the socioeconomic factors: age; income; education level; gender; housing type; population density; country of origin; and employment level. The results show that decreases in public transport use are linked to all these factors.

The second model groups the investigated areas into five distinct clusters based on the socioeconomic data, showing the impacts for different socioeconomic groups. During the autumn the differences between the groups diminished, and especially Cluster 1 (with the lowest education levels, lowest income and highest share of immigrants) reduced their public transport use to a similar level as the more affluent clusters.

**Results:**

The results show that socioeconomic status affect the change in behaviour during the pandemic and that exposure to the virus is determined by citizens’ socioeconomic class. Furthermore, the results can guide policy into tailoring public transport supply to where the need is, instead of assuming that e.g. crowding is equally distributed within the public transport system in the event of a pandemic.

**Supplementary Information:**

The online version contains supplementary material available at 10.1186/s12544-021-00488-0.

## Introduction

COVID-19 has severely affected the world with increased mortality rates, stagnating economies and isolated citizens. Over 1.3 million deaths were reported by the 24th of November [39]. The International Monetary Fund estimates that the economy will shrink by 4.4% in 2020 [13]. However, the impacts of the virus are unequally distributed, both across the world and within countries and regions [10, 19, 26]. The number of cases locally in Stockholm has also varied substantially with more cases in impoverished neighbourhoods than affluent neighbourhoods [21, 28].

Public transport is one of the sources of virus transmission [37], and its use has therefore been suspended or limited by either restrictions or voluntary measures [29]. Still, many citizens rely on public transport as their primary or only means of transport, resulting in both further spread of the virus and in inequality of risk [7, 19]. In order to combat the spread and the associated inequalities it is adamant that we increase our understanding of which citizens continue to use public transport and plan proactively.

Research on COVID-19 is moving quickly. To the best of our knowledge, studies in the transport domain have mainly been concerned with either:

1. Measuring the magnitude of the overall decrease in travel [9, 20, 22, 25, 42].
2. Measuring or simulating the spread of the virus through transportation [24, 27, 41].
3. Assessing the effectiveness of mobility restrictions [4, 18, 29, 38].

A few studies have investigated behavioural changes and linked them to income [6, 14, 17, 41], ethnicity [17, 19, 41], psychological traits [3] and political viewpoints [17], using either surveys (with risk of biased or small samples) or cell phone data (with no or little information on transport mode). However, to the best of our knowledge, no previous studies have attempted to comprehensively study the relations between socioeconomic factors and citizens’ change in public transport utilization during the pandemic.

**Fig. 1:**
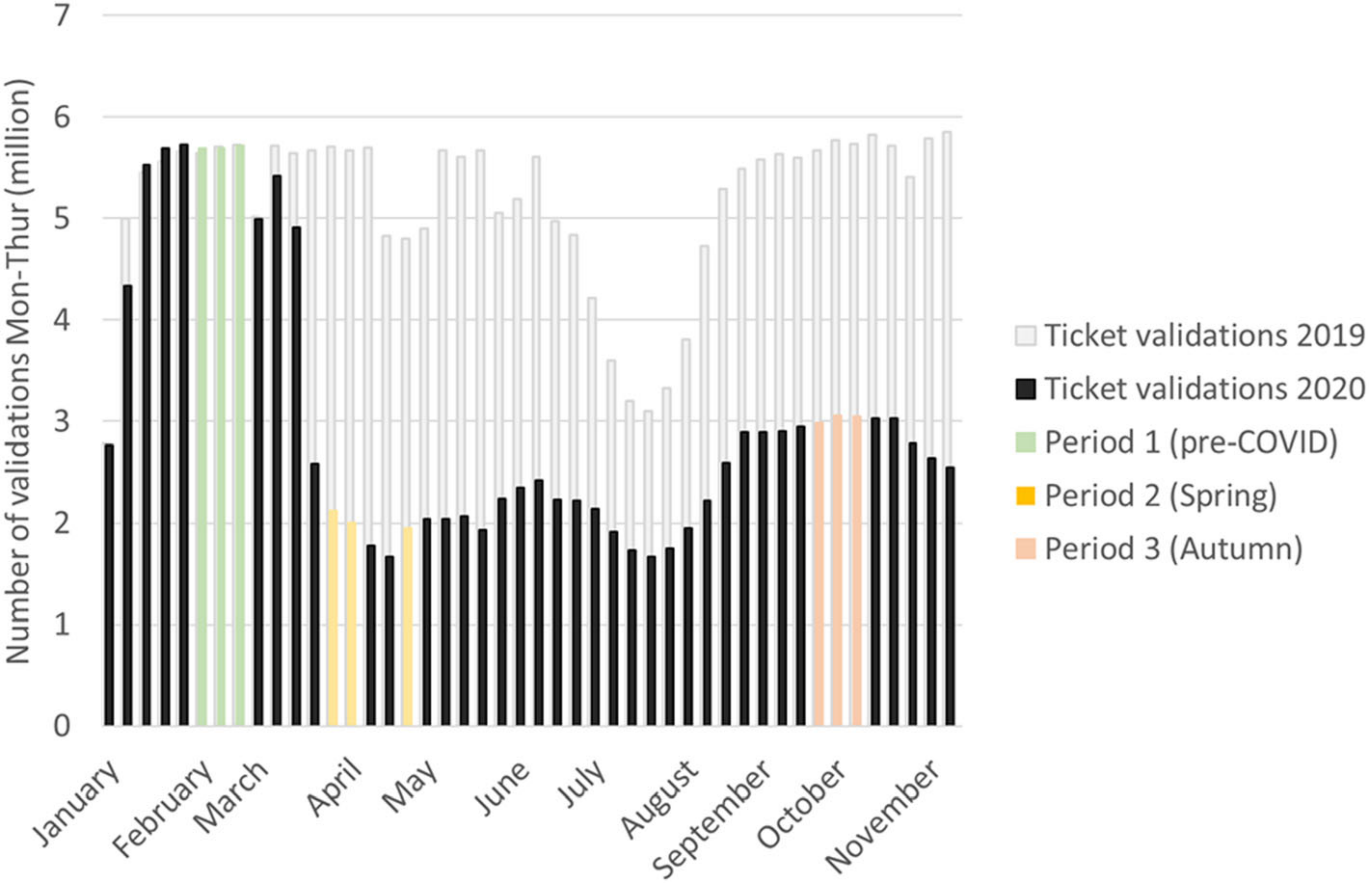
Number of validations per week (Monday – Thursday) and description of the three chosen periods featured in the paper. For context, ticket validations for 2019 (grey) and 2020 (black) are featured. Period 1 (yellow) is used as a reference period while period 2 (pink) evaluates the effects during spring and period 3 (blue) the effects during autumn. Period 2 is divided into two due to the Easter holiday occurring during 2 weeks

In this study, we assess the relationship between socioeconomic factors and change in individuals’ public transport use during COVID-19 in Stockholm, Sweden. Specifically, we consider whether each card was actively used during each of two periods, with a period before the outbreak as a reference. We denote the reference period as Period 1 (February 2020) and the two periods during the pandemic as Period 2 (23rd of March to 20th of April 2020, “Spring”) and Period 3 (14th of September to 11th of October 2020, “Autumn”), respectively. Figure 1 shows an overview of the periods.

The dataset consists of ticket validations from 1.8 million individual smart cards with persistent and unique identification numbers, allowing us to investigate the change in travel behaviour of (anonymous) individuals. We correlate this anonymous travel activity during COVID-19 with demographic data on 1287 areas in Stockholm County, establishing links to common social factors such as gender and age. Furthermore, we develop a framework using clustering of the socioeconomic dataset to enhance the understanding of the changes for different social groups within Stockholm.

As the change in ridership tends to be unevenly distributed in the public transport systems, there should be a potential to more effectively use the capacity in places where the demand is still prevalent. A greater understanding of how different citizens are changing their behaviour is therefore paramount to combat the spread of COVID-19 through public transport, all the while providing sufficient travel options for those that need to travel. Our findings provide insights to policy makers and planners in understanding how the virus spreads, the design of equitable responses to a pandemic and the creation of proactive strategies for the future, with socioeconomic markers commonly used by planners across the globe.

The remainder of the paper is organized as follows. Section 2 describes the method used to extract travel behaviour data from smart card ticket validations and to analyse the impacts of socioeconomic factors. Section 3 introduces the settings of the study, followed by results presented and discussed in Section 4. Section 5 concludes and deliberates on policy implications of the findings. The complete result tables with e.g. confidence intervals of the two models can be found within the Additional file 1.

## Data and methods

**Fig. 2:**
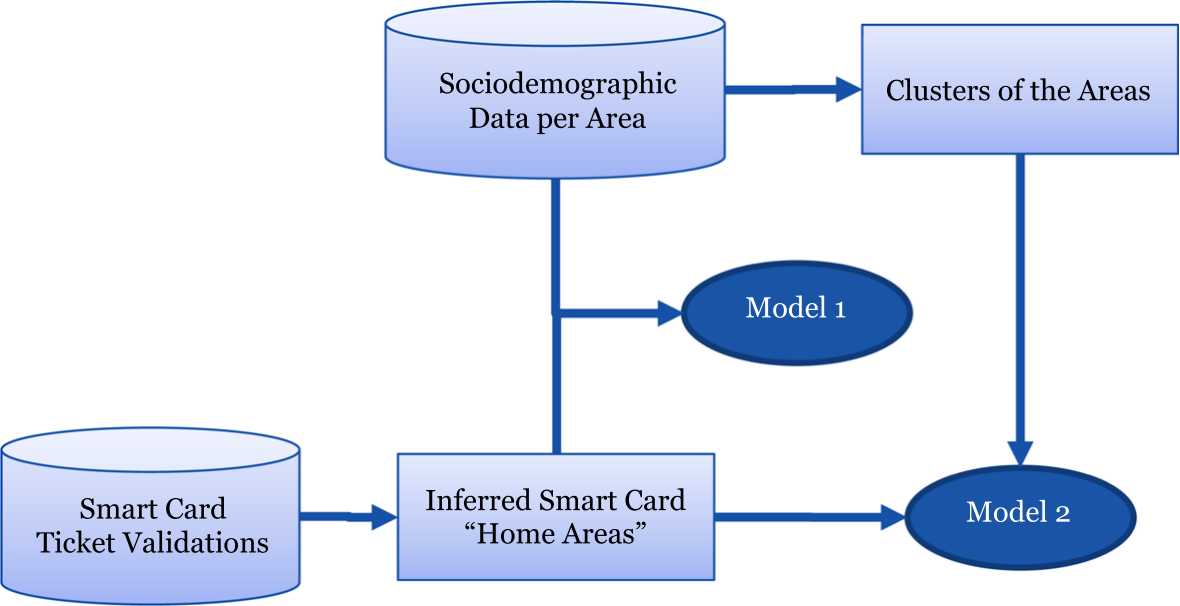
Overview of the analysis methodology

This study uses two datasets: smart card ticket validation data on an individual card level and sociodemographic data divided into 1287 areas for Stockholm County, Sweden. We propose two binomial logit models for understanding the results, Model 1 which examines each sociodemographic variable (e.g. income) separately and Model 2, which establishes a cluster analysis to more easily understand the impacts for different social groups within the region. An overview of the process is shown in Fig. 2.

This section is divided into four subsections: Section 2.1 describes how the smart card dataset is linked to geographical areas within the county; Section 2.2 describes the sociodemographic dataset and how we have established the five clusters; and Section 2.3 outlines the binomial logit model used for both Model 1 and Model 2.

### Inferring travel statistics and home areas from validation data

The ticket validation system used by Stockholm County public transport is a tap-in only system, which means that the data do not contain exit points (tap-outs) for any trips. To remedy this, a framework was developed for inferring complete trip travel diaries for individual cards (see Cats et al. [2] for a comprehensive overview of the framework). The travel diaries are used to connect consecutive tap-ins to trips (journey segments between transfers) and journeys (from origin stop to destination stop including transfers), and to infer the home location of each card holder.

In summary, the framework infers the alighting location of a trip *k* by searching within a maximum defined radius (e.g. 1 km) around the next tap-in *k + 1* location (if recorded within the five following days). The trip’s alighting location is inferred as the closest alighting location that also matches one of the transit lines or modes from the *k* tap-in. Otherwise, the tap-out location to tap-in *k* is not inferred. For example, a tap-in at station A in the morning followed by a tap-in at station B in the afternoon would indicate that the first trip made was from station A to station B.

Similar inference frameworks have been developed with good results (see for example Seaborn et al. [30], Trépanier et al. [36] and Zhao et al. [43]). The framework can be biased towards frequent commuting and might fail to infer tap-out locations of very infrequent travellers as we have used a five-day rule when the next tap-in could be considered. Furthermore, the framework has a number of known caveats, such as measuring travel on trams or light rail where only a fraction of passengers validate their ticket.

Each card’s home area is also inferred. For each card, the algorithm counts the number of days when the first journey of the day takes place from a particular area. The area with the highest count during the Pre-COVID period is classified as the home location for the card. The socioeconomic data are linked to the individual travel pattern data based on the statistics area that comprises each card’s most prevalent morning tap-in. Note that while the smart-card data are individual, the socioeconomic information is aggregated for an area, making it more descriptive of the type of neighbourhood the traveller comes from rather than their individual socioeconomic status.

The study uses an almost full set of public transport smart card and mobile app tickets in Stockholm County.^1^ Considering the period of January 1–February 29, 2020, the framework infers tap-out locations for 90.2% of Stockholm County tap-ins. 75.1% of tap-ins have also inferred vehicle and travel time, which is 83.2% of all trips. 86.2% of the journeys have both a destination location and travel distance known. For each of the 1.8 million cards/devices, statistics are collected for both the Pre-COVID and the COVID periods. These statistics include the number of active days, number of trips, number of journeys, modes used, the average number of trips and journeys per active day, and used ticket type.

The data have some limitations due to the pandemic. From March 17 a safety policy was implemented in Stockholm where bus passengers were directed to enter only through the back doors. The measure was taken to protect the drivers and make distancing easier for the passengers. However, validation terminals are not present at the back doors for almost all buses in the system. Therefore, bus trips do not show up in the data. To remedy this we exclude cards used exclusively for bus trips in both Pre-COVID and COVID periods. Fortunately, only about 94% of the cards are used in several modes of transport (e.g. a bus trip followed by a transfer to the metro) and would therefore show up as “active” during the COVID periods. The assumption is that these individuals still live in the home area inferred from the Pre-COVID period.

### Clustering of areas based on socioeconomics

In our alternative model, we use multidimensional social classes instead of individual socioeconomic variables. This approach might, especially for those with local knowledge, improve the ability to make sense of the data, and understand the combined effect of many different socioeconomic factors. Therefore, we cluster the areas by socioeconomic variables to construct the new independent social class variables used in model 2. Clustering as a method is mostly used within the marketing field as a technique to understand customers but is also used within varying fields such as automation and transportation [11].

We implement a two-step cluster analysis algorithm using the statistical software SPSS [12]. This approach automatically selects the natural number of clusters in the dataset by finding the increase in the distance between the two closest clusters across all stages of hierarchical clustering. The method is designed for large datasets that involve initial pre-clustering aggregation in the first step. Furthermore, it can handle both categorical and continuous variables. These attributes make two-step cluster analysis a good candidate for finding the natural number of area types in Stockholm County.

Area statistics from Statistics Sweden on 1287 demographic statistical areas of Stockholm County (DeSO) are used for this study [31]. The socioeconomic data comprise eight categories: housing, education, income, gender, age, country of birth, unemployment and area population density as shown in Tables 1 and 2 (see Sections 3.3 and 3.4). The data are linked to the individual travel pattern data using the DeSO areas that comprises each card’s most prevalent morning tap-in location during the Pre-COVID period.

**Table 1:** Description of socioeconomic data (further details are available at ([31], in Swedish))

Name	Possible values (share of the total population for the county)	Comment
**Housing type**	Cooperative apartment (35%) Owned housing (31%) Rented housing (31%)	Cooperative apartments are a common way of owning your apartment in Sweden. Formally, however, the household purchases a share of a property. The dataset does not contain any information on 3% of the population.
**Education**	Less than Upper secondary school educated (10%) Upper secondary school educated (35%) University educated (52%)	The dataset does not contain any information on 3% of the population. The original dataset categorises the university education into two steps, while we have merged them.
**Income**	Income below median (50%) Income above median (50%)	Share of the area population (age 20 and above) who have an income below or above Stockholm Region median income.
**Population density**	Sparsely populated Semi densely populated Densely populated	Classification by Statistics Sweden.
**Age**	< 20 years old (24%) 20–29 years old (13%) 30–39 years old (15%) 40–64 years old (31%) > 65 years old (16%)	The original dataset divides the age groups into ~ 5-year classes (i.e. 20–24, 25–29). For simplification purposes we merged them into five classes
**Country of birth**	Born in Sweden (66%) Abroad (34%)	Note that the Abroad groups is quite diverse, with both people who have immigrated as refugees as well as e.g. labour migration.
**Employment**	Employed (80%) Non-employed (20%)	Note that this variable does not indicate only unemployment, but ‘non-employed’ includes e.g. students and retirees. The variable only describes the population of 20–64 year olds (most people retire at 65).
**Gender**	Male (50%) Female (50%)

**Table 2:**
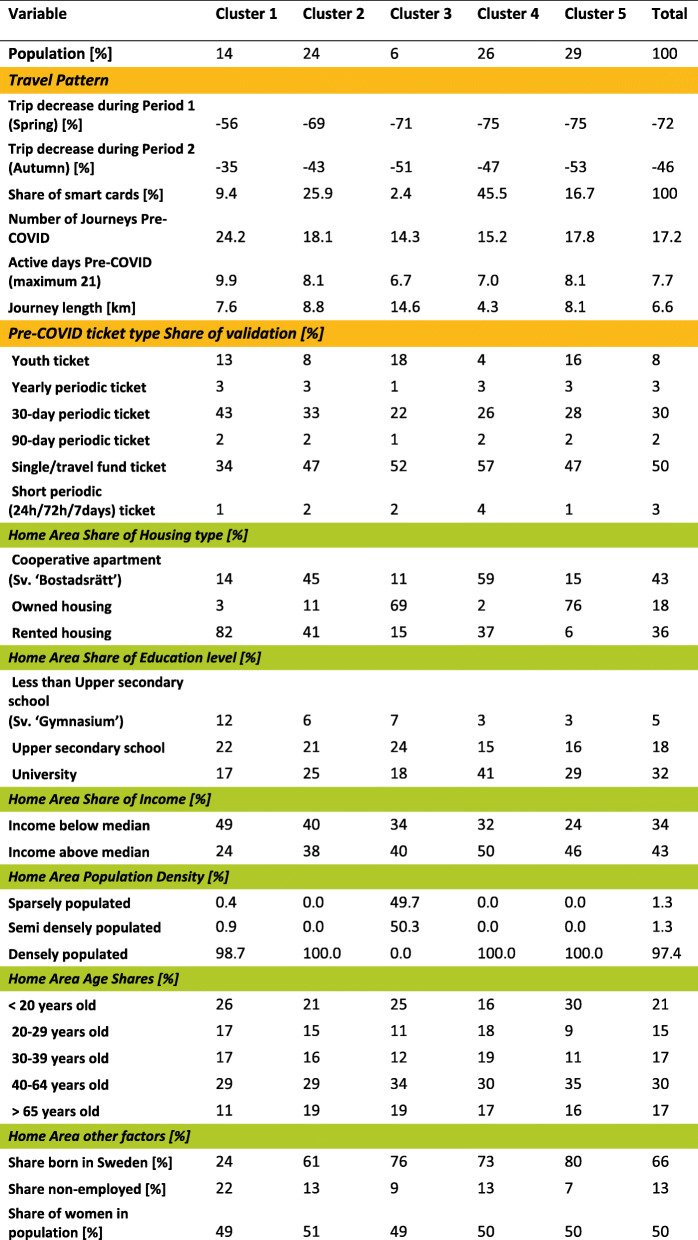
The full dataset has 1.8 million individual cards, where each card has an origin zone, and each zone is designated to one of the five clusters. This tabulation describes the average card, and its origin zone attributes, from each of the five clusters and the full set. Travel patterns and ticket types are shown as yellow and socioeconomic variables as green

### Binomial logit model

The decrease in travel during COVID-19 is measured by the reduction of trips for each individual card holder. The dependent variable is constructed as having one of two values: 1 in the case of trips decreasing with more than 90% from Pre-COVID (Period 1) to COVID (Period 2 or 3, respectively), and 0 otherwise. The binomial logit model is chosen given the dependent variable’s binary nature [5]. The dependent variable is thus expressed as a function of the utility *U*_*i*_ *= V*_*i*_ *+ ε*_*i*_, where *V*_*i*_ and *ε*_*i*_ are the deterministic and random parts of the utility, respectively. The probability of a substantial trip decrease is then

1 $$ \mathit{\Pr}=\frac{1}{1+{e}^{V_i}} $$

The utility *V*_*i*_ is a linear function of estimated parameters (*β* and *γ*) and explanatory variables describing the individual’s situation,

2 $$ {V}_i={\sum}_j{\beta}_j{x}_{ij}^{AC}+{\sum}_k{\gamma}_k{x}_{ik}^{HA} $$

These variables pertain to individual cards activity Pre-COVID (*AC*) and the socioeconomic composition of the cards home-area (*HA*). Model 1 is modelled with *HA* comprising of each socioeconomic variable (e.g. income and age), whereas Model 2 uses the five different clusters established in Section 3.4.

## Case study

### Stockholm public transport

Stockholm County has approximately 2 million inhabitants, and about 30% of all trips within the county are made by public transport. Public transport is far-reaching but mainly tailored to serving commute trips to the central parts of the county where it offers a high level of service, and travel times are shorter than for the corresponding car trip. For trips between the inner city and the surrounding county, public transport boasts a 70% mode share. Within the inner city, the mode share is about 40% (walking and bicycling constitute another 40%, although varying depending on the season and daily weather), compared to a share for public transport of less than 25% in the outer parts of the region [33].

### The local context of COVID-19

Sweden has been heavily affected by the pandemic in terms of deaths/capita [39]. Sweden early on chose a path of voluntary measures for citizens to abide by, instead of the more common approach of many countries with mandatory curfew or lockdowns [29]. These voluntary measures have had the effect of decreased travel but with high variance. Air traffic had decreased by around 80% [34], but car traffic on national highways by only around 15% [35] as of November 2020.

During spring 2020 the Public Health Agency advised against the use of public transport, limiting social contacts (especially for the elderly) and avoiding travelling if not necessary. Upper secondary schools and universities were closed in exchange for remote teaching and a large part of the population started to work from home, especially in Stockholm county which experienced large impacts during the early phases of the pandemic. The guidelines were relaxed during the summer, but still recommended remote working and to avoid unnecessary travel, especially with public transport. During late October, the tougher guidelines were imposed again as a consequence of increased spread of the pandemic within Sweden and Stockholm.

Public transport ridership fell dramatically during March and recovered somewhat during the summer, followed by a relatively stable autumn (see Fig. 1) until October when stricter guidelines were posted by the Public Health Agency. Meanwhile, apart from a temporary reduction in bus services from late March to early May, supply (in terms of departures and seat capacity per hour) was held relatively constant during the spring and autumn. The reduction in ridership was thus in general not caused by reduced levels of service. For a more comprehensive timeline and the impacts on ridership, see Jenelius [15] and Jenelius and Cebecauer [16].

### Area statistics

The socioeconomic dataset used in this study, DeSO, is publicly available from Statistics Sweden ([31], website and data in Swedish). The dataset is provided per area and consists of eight common variables for describing the composition of the population: Gender, Age, Education level, Income, Country of origin, Employment level and Housing type. We have edited the dataset lightly to simplify the analysis, most notably is the aggregation of the age groups, from 17 groups to 5. The dataset is listed in Table 1.

### Area clusters

The clustering of home areas is conducted based on the eight categories of socioeconomic data. Five distinct clusters are identified (Silhouette measure of cohesion and separation was 0.4):

1. Cluster 1. Low income and education levels, high levels of unemployment and large shares of children and residents born outside of Sweden. The areas are spread out within the county, primarily in parts with high share of rental apartments.
2. Cluster 2. Slightly higher income, less unemployment, and fewer residents born outside of Sweden. This group is the one most spread out across different parts of the county.
3. Cluster 3. Focused on more rural parts of Stockholm County where people own their homes, have more children and most residents were born in Sweden. This group has medium level incomes but less education than the average for the county.
4. Cluster 4. Focused on more central parts of the county with high income and education and the largest share of 30–39-year-olds.
5. Cluster 5. Live in the so called “garden suburbs”^2^ close to the city centre with homeowners and high incomes and education levels.

The dataset with each card’s travel patterns, ticket types, and origin zone is adjoined with the origin zones’ socioeconomic statistics and cluster type. Table 2 presents the card dataset as average values for cards in the five clusters and average values for the full data set.

Three of the groups have similar population sizes while Cluster 1 and 3 are smaller than the others. People within Cluster 3 also travel less with public transport per person than other groups. More precisely, those that travel make fewer journeys and are active fewer days. The studied Pre-COVID period is 3 weeks, so a person commuting each day with public transport on weekdays would make about 30 journeys during the period (circa 2 journeys per weekday). Most of the users in Cluster 1 seem to be this type of public transport user.

**Fig. 3:**
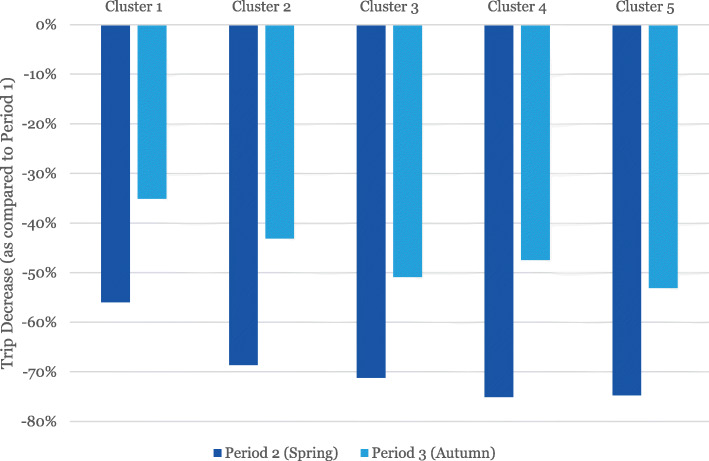
The decrease in public transport trips for the five identified clusters compared to the reference period

Cluster 4 is the most educated, while Cluster 5 is the group with the highest income. Income distributions between Cluster 5 and 1 are nearly reversed, with the majority above-median income in the former group and below in the latter. The two poorest groups also have the lowest levels of residents born in Sweden. Residents in Cluster 3 almost exclusively live outside the areas classified as densely populated. Apart from Cluster 1 having a slightly younger population, all groups have a similar age distribution. The share of non-employed is the highest in Cluster 1 (Fig. 3).

Owned housing typically means one-household houses, while cooperative apartments are flats where the owner co-owns a share in the apartment building proportional to the apartment’s size (Swedish “Bostadsrätt”). Cluster 5 has the highest percentage of owned housing and Cluster 4 has the highest percentage of cooperative apartments, while Cluster 1 has a substantial majority of rented apartments.

## Results and discussion

To understand the driving factors behind the decrease in public transport use during the pandemic, we estimate two binomial logit models as described in Section 2.3. Model 1 uses each separate factor such as age and income as independent variables, while Model 2 instead uses the five clusters as independent variables. Both models use Pre-COVID travel and ticket information as independent variables while the dependent variable is defined as the share of individual cards that has a major decrease (<− 90%) in trips during spring and autumn compared with the Pre-COVID period. The independent variables for the two models are indicated in Table 3.

**Table 3:** Included variables in models 1 and 2

Variable	Model	Description
**Number of journeys pre-COVID**	1, 2	Number of journeys logged by each individual card
**Number of active days pre-COVID**	1, 2	Number of days when the individual card have been used
**30 Days Travel Card**	1, 2	Share of journeys made during pre-COVID using this ticket type
**90 Days Travel Card**	1, 2	“
**Single journey ticket**	1, 2	“
**Yearly Travel Card**	1, 2	“
**Visitor Travel Card**	1, 2	“
**Youth Travel Card**	1, 2	“
**Share - Owned housing**	1	Share of population in the individual cards home area living in this housing type
**Share - Cooperative apartment**	1	“
**Share - Rented housing**	1	“
**Share - University educated**	1	Share of population in the individual cards home area with this education level
**Share - Upper secondary school**	1	“
**Share - Less than Upper secondary school**	1	“
**Share - Income above median**	1	Share of population in the individual cards home area with income above the county median
**Share - Income below median**	1	Share of population in the individual cards home area with income below the county median
**Share - Born in Sweden**	1	Share of population in the individual cards home area that are born in Sweden
**Share - 20-29 years old**	1	Share of population in the individual cards home area within this age bracket
**Share - 30-39 years old**	1	“
**Share - 40-64 years old**	1	“
**Share - > 64 years old**	1	“
**Share - Non-employed**	1	Share of population in the individual cards home area that are unemployed
**Share - Female**	1	Share of population in the individual cards home area identified as women
**Cluster 5**	2	Categorical variable defining the individual cards home area
**Cluster 4**	2	“
**Cluster 3**	2	“
**Cluster 2**	2	“
**Cluster 1**	2	“
**Sparsely populated**	1	Categorical variable defining the individual cards home areas population density
**Semi densely populated**	1	“
**Densely populated**	1	“

**Fig. 4:**
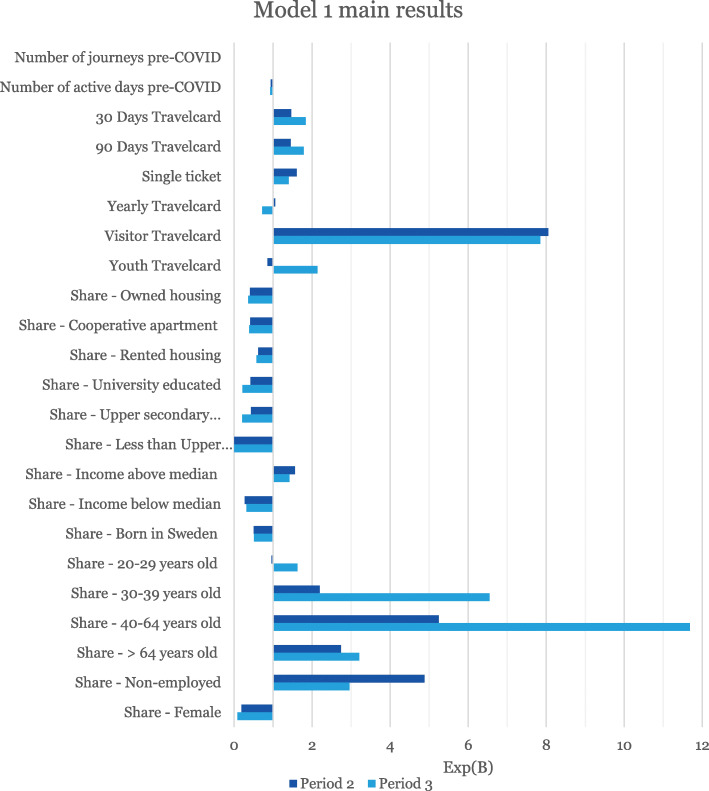
The main results of Model 1 estimations. All parameters are significant at the 5% level, except the Share of 20–29 year olds for Period 1. Note the difference in scale between Figs. 4 and 5. See the Additional file 1 for details

**Fig. 5:**
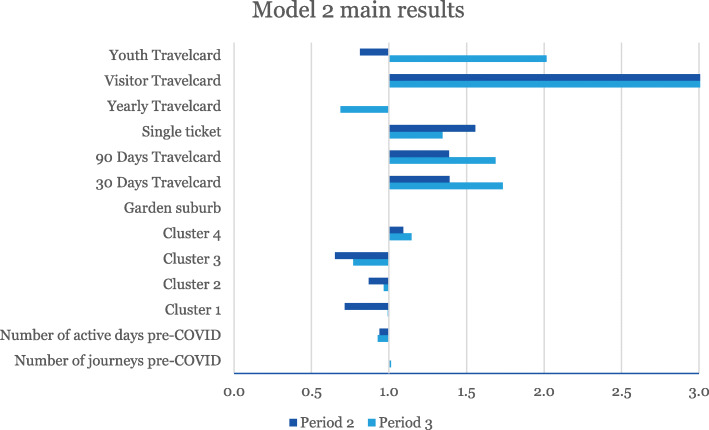
The main results of Model 2 estimations. The values for Visitor Travel card (8.0 for Period 2 and 7.7 for Period 3) are truncated in order to better represent the other factors used. All parameters are significant at the 5% level, except for the Yearly Travel card for Period 2 and Cluster 1 for Period 3. All factors have small confidence intervals, except the variable for Visitor Travel card for period 2. Note the difference in scale between Figs. 4 and 5. Please see the Additional file 1 for details

The overall results from Models 1 and 2 are presented in Figs. 4 and 5, respectively. Full model estimation results are available in the Additional file 1, including parameter standard errors and confidence intervals.

Exp(B), also called the *odds ratio*, indicates the relative change in the odds of substantially reducing public transport use given a one-unit increase of a particular explanatory variable while other variables are held fixed. Thus, a value greater than one indicates a positive effect on the probability to stop travelling, and a value smaller than one means a negative effect. For example, Fig. 4 indicates that zones with a large share of 30–39 year olds have a high propensity to decrease their travel frequency.

Several variables represent shares of area populations, e.g. housing conditions. These numbers should primarily be interpreted in relation to other categories within the same factor. For example, in the case of housing, smart card holders in areas with a large share of rented housing have decreased their public transport use to a smaller extent than in areas with large shares owned housing or cooperative apartments.

The age factor for Model 1 uses 0–19 year olds as reference category. For example, this means that all other age groups decreased their public transport travelling to a larger extent than the 0–19 year olds for Period 3, while there were no significant difference between 20 and 29 year olds and 0–19 year olds in Period 2. Model 2 uses Cluster 5 as the reference cluster.

The following subsections expands on the impacts of different groups of variables. For further details, please see the Additional file 1.

### Model 1 – separate socioeconomic factors explaining public transport travel decreases

Almost all variables in Model 1 are significant, except for the share of 20–29 year olds for Period 2 (spring). The results indicate that socioeconomic factors influence people’s change in behaviour when it comes to public transport use and furthermore, that the type of card used pre-COVID contributes to explaining the change in public transport use. In general, the results for the spring and autumn periods are similar, with a few factors changing sign (e.g. Youth Travel cards) and some factors fluctuating substantially (e.g. the age groups).

The impact of the age-related explanatory variables for Period 2 (spring) confirms our expectations that seniors avoided public transport (odds ratio 2.7), although the variation seems to be large (see the comparatively large confidence intervals in Table S1 in the Additional file 1). The differences among age groups are even more noticeable for Period 3 (autumn), which may be linked to many schools being closed during the spring but open during the autumn. Similar to our findings, Chan et al. [3], found age to be an explanatory factor of travel during COVID-19, while Kavanagh et al. [17] on the other hand found no connection between mobility and age. It should, however, be noted that Chan et al. [3] and Kavanagh et al. [17] investigate all modes of travel while we focus on public transport.

Similarly, residing in an area with high income and education is correlated with reduced public transport use. These results confirms previous findings by Dahlberg et al. [6], Jay et al. [14], Kavanagh et al. [17], WSP Sverige AB [40] and Yechezkel et al. [41]. However, our results show overlapping confidence intervals between the two groups with an education level of at least Upper secondary level (Swedish “Gymnasium”) while the group with the lowest education level had a higher propensity to continue using public transport.

We also find that gender influences the results. The probability to stop travelling by public transport increases with the share of the male population in the area. This finding is similar to the results of Molloy et al. [23] but contrasted by Chan et al. [3] and Kavanagh et al. [17]. Again, these three previous studies investigated mobility overall, without respect to mode. The reason for this variation is however difficult to assess and needs more research.

Travellers in areas with large immigrant populations were more likely to reduce their public transport travelling when we account for factors such as education level, income and unemployment. This effect is reversed compared to the conclusions of Kavanagh et al. [17] and Yechezkel et al. [41]. Immigration is connected to lower income, education and employment levels [1] and immigrants tend therefore to travel more by public transport, but our findings indicate that this is mainly due to other socioeconomic factors. It should, however, be noted that ethnicity and birth-country are not interchangeable traits and that the immigrant population of Sweden is quite heterogeneous [32], so the reason for immigrant populations to decrease their public transport use more than natives needs further examination.

Travellers from areas with many non-employed tend to go back to using public transport to a larger extent during the autumn. We have not found independent corroboration of this tendency, but testing a model formulation without the Non-employed variable yielded reasonable effects: the effect of the non-employed variable is distributed on corresponding age groups (20–64), education groups (larger effect with lower education) and income (larger effect for low income).

The type of ticket used also contributes to explain some of the changes. Unsurprisingly, people using Visitor Travel cards drastically reduced their public transport trips during both spring (odds ratio 8.1) and autumn (odds ratio 7.8) periods.

Individuals using the Youth Travel card decreased their public transport trips to a lesser extent than the other groups for Period 2 (odds ratio 0.9), while the results for Period 3 (odds ratio 2.1) indicate that they to a large extent reduced their public transport trips. This finding is unexpected as Upper Secondary schools (attended by mainly 16–19 year olds) were closed down during spring but remained open during the autumn, which should have resulted in reversed signs (due to the reduced need of travelling by public transport). One hypothesis, which we have seen anecdotal evidence of, is that adolescents may have shifted to the “free” bus alternative (all controls of tickets for buses were suspended, meaning few repercussions for travelling without a ticket). However, we would like to stress that this hypothesis is speculative and needs further investigation.

### Model 2 – using socioeconomic clusters to explain reduced public transport travel

Similar to Model 1, almost all variables in Model 2 are significant except for the Yearly Travel card for Period 2 and Cluster 1 for Period 3. The variables related to Travel card type have similar effects as for Model 1, while the effects of the clusters vary more than for Model 1. Note that Cluster 5 acts as the reference value for the clusters.

The people living within Cluster 3 areas continued to travel to the largest extent (odds ratio 0.64 in Period 2, 0.75 in Period 3). Overall, public transport ridership is lower in rural areas due to lower relative attractiveness in relation to the car. It is therefore likely that people utilizing public transport may have few other options. Most trips are also long, more than 10 km, making walking and cycling unviable (see Table 2).

Cluster 1 is, after Cluster 3, the group that continued to use public transport to the largest extent during Period 2 (odds ratio 0.71). This is the cluster with the lowest education and income and with the highest unemployment together with a big immigrant population and most likely a group with low car ownership rates (unfortunately not part of the dataset). However, this cluster experienced the largest change between the two periods.

Cluster 2 has a somewhat similar effect as the Cluster 3, albeit with smaller magnitude (odds ratio 0.87 in Period 2), and a result more similar to Cluster 5 cluster for Period 3 (odds ratio 0.97).

Finally, inhabitants within Cluster 4 decreased their public transport travel the most (odds ratio 1.08 in Period 2), with an increased tendency during Period 3 (odds ratio 1.15). This group most likely has walking and bicycling as viable options since most trips tend to be short (4.3 km on average).

Overall, the differences between the clusters diminished between Period 2 and Period 3, which may be due to society’s adaption to the crisis. The recommendations were relaxed somewhat, which may indicate that some public transport travellers from the groups who had previously telecommuted (correlated to high income levels according to Dingel and Neiman [8]) increased their public transport use.

## Conclusion

This study has shown that there is substantial variation in the decreased use of public transport during the ongoing pandemic. Furthermore, we have clustered the socioeconomic dataset into five distinct clusters, highlighting how the pandemic has influenced people with different social backgrounds. The results show that those with the least resources have continued travelling with public transport to the greatest extent, creating a connection between wealth and risk of exposure to a potentially fatal disease. However, this variance seems to have decreased over time.

The findings have important implications regarding the equity aspects, how we model the spread of diseases and the response from public transport authorities. Several other authors (e.g. Kavanagh et al. [17], Laurencin and McClinton [19] or Prats-Uribe et al. [26]) have established the disparities related to both transport and deaths during the pandemic. The virus itself may discriminate by age, but a decent society should give all citizens equal opportunities to avoid the spread of the infection. This inequality is directly related to several of the United Nations sustainability goals on equality and healthcare. Public transport may be one source of transmission of the disease, but as we have shown, the risk of catching the virus through public transport varies substantially.

Since the socioeconomic variables are commonly available data, the methodology should be transferable to other regions. Although the context is 1) specific to a pandemic, 2) the policies implemented in Stockholm and 3) the cultural and therefore behavioural response, we have highlighted the socioeconomic variables that impact a citizen’s inability to stop travelling by public transport. It should be feasible to adjust public transport models to account for the demand change given our findings in other settings, and although the model may not yield a perfect result, it should still indicate where the need for public transport is the greatest. Furthermore, models for how the diseases spreads through society need to adhere to that not all social groups are equally likely to catch the virus through public transport, and our findings could contribute to better viral dispersion models.

A pressing problem for all public transport administrations is to keep the speed of contagion down. One obvious way of doing this is to reduce the demand as much as possible, minimising the interaction between the passengers. The analysis shows that the demand for public transport varies substantially, and supply should vary accordingly in order to minimise the number of passengers per vehicle, and not just the total average. In particular, supply should be rerouted to more impoverished neighbourhoods and decreased in the more affluent areas.

Authorities should also consider directing information regarding how to behave in public transport towards those more likely to use public transport, e.g. those with lower income or education level. Changed travel patterns should prompt the public transport authorities to rethink the range of ticket types offered to the public. In the semi-stable situation, as during the autumn, many travellers with the option to telecommute might have chosen to travel to work 1 or 2 days in the week using single journey tickets. This travel pattern change would explain the shift in odds ratio between 30 Days Travel Card and Single Ticket for our two periods (higher odds ratio for the Single ticket in the spring and the Periodic ticket in the autumn). To salvage revenue in that situation, the authorities might introduce low-frequency periodic tickets with a limited number of journeys per week and a lower price than the regular periodic cards.

## Supplementary Information

**Additional file 1: Table S1.** Results from model 1 for Period 2 (spring) for Public transport travel patterns (yellow) and socioeconomic data (green). **Table S2.** Results from model 1 for Period 3 (autumn) for Public transport travel patterns (yellow) and socioeconomic data (green). **Table S3.** Results from model 2 for Period 2 (spring) for Public transport travel patterns (yellow) and the clusters created from the socioeconomic data (green). **Table S4.** Results from model 2 for Period 3 (autumn) for Public transport travel patterns (yellow) and the clusters created from the socioeconomic data (green).

## Data Availability

DeSO data is publicly available data provided by Statistics Sweden at: https://www.scb.se/hitta-statistik/regional-statistik-och-kartor/regionala-indelningar/deso%2D%2D-demografiska-statistikomraden/ (Accessed 2020-06-05, website in Swedish). Data on travel patterns is the proprietary data of Region Stockholm. Region Stockholm have given their consent for the use of the dataset for this study. The datasets analysed during the current study may be available for other researchers from the corresponding author, conditioned by Region Stockholm.

## Abbreviations

DeSO: Demografiska statistikområden (our translation: ‘Demographic statistical areas’)
Period 1: Also referred to as the Pre-COVID period, between February 3rd and 26th 2020
Period 2: Also referred to as the Spring period, between March 23rd and April 5th and between April 20th and 26th 2020
Period 3: Also referred to as the Autumn period, between September 14th and October 11th 2020

## Footnotes

1 A small share of passengers travel for free, e.g. children under the age of 7. This share is estimated to be less than 5%.

2 See https://en.wikipedia.org/wiki/Garden_city_movement#Garden_suburbs for an international comparison, however the Swedish concept is mainly targeted towards the middle class.
