# Supplementary material for: Who continued travelling by public transport during COVID-19? Socioeconomic factors explaining travel behaviour in Stockholm 2020 based on smart card data
Source: Eur Transp Res Rev. 2021 Jun 7;13(1):31. doi: 10.1186/s12544-021-00488-0 (PMC8180438; doi:10.1186/s12544-021-00488-0)
Supplement: Supplementary file 1 — Additional file 1: Table S1. Results from model 1 for Period 2 (spring) for Public transport travel patterns (yellow) and socioeconomic data (green). Table S2. Results from model 1 for Period 3 (autumn) for Public transport travel patterns (yellow) and socioeconomic data (green). Table S3. Results from model 2 for Period 2 (spring) for Public transport travel patterns (yellow) and the clusters created from the socioeconomic data (green). Table S4. Results from model 2 for Period 3 (autumn) for Public transport travel patterns (yellow) and the clusters created from the socioeconomic data (green). [file 12544_2021_488_MOESM1_ESM.docx]

1. Additional file 1

Tables S1-S4 show the output of the binominal logit model, where the Exp(B) value being presented in Figures 4 and 5 in the main body of the article.

- 1. Model 1

Table S1. Results from model 1 for Period 2 (spring) for Public transport travel patterns (yellow) and socioeconomic data (green).

| Period 2 | | | | | | | | | |
| --- | --- | --- | --- | --- | --- | --- | --- | --- | --- |
|  | B | S.E. | Wald | | df | Sig. | Exp(B) | 95% C.I.for Exp(B) | |
|  |  |  |  |  |  |  |  | Lower | Upper |
| Travel Pattern | | | | | | | | | |
| Number of journeys pre-COVID | -0.004 | 0.000 | 181.404 | 1 | | 0.000 | 0.996 | 0.996 | 0.997 |
| Number of active days pre-COVID | -0.063 | 0.001 | 6301.608 | 1 | | 0.000 | 0.939 | 0.938 | 0.941 |
|  |  |  |  |  | |  |  |  |  |
| Ticket type | | | | | | | | | |
| 30 Days Travel card | 0.386 | 0.014 | 790.998 | 1 | | 0.000 | 1.471 | 1.432 | 1.512 |
| 90 Days Travel card | 0.373 | 0.016 | 532.079 | 1 | | 0.000 | 1.5 | 1.407 | 1.499 |
| Single ticket | 0.472 | 0.013 | 1236.455 | 1 | | 0.000 | 1.6 | 1.562 | 1.646 |
| Yearly Travel card | 0.054 | 0.016 | 11.778 | 1 | | 0.001 | 1.1 | 1.023 | 1.089 |
| Visitor Travel card | 2.086 | 0.021 | 9830.925 | 1 | | 0.000 | 8.1 | 7.727 | 8.391 |
| Youth Travel card | -0.153 | 0.014 | 113.565 | 1 | | 0.000 | 0.9 | 0.835 | 0.883 |
|  |  |  |  |  | |  |  |  |  |
| Housing conditions | | | | | | | | | |
| Share - Owned housing | -0.898 | 0.053 | 284.059 | 1 | | 0.000 | 0.4 | 0.367 | 0.452 |
| Share - Cooperative apartment | -0.878 | 0.053 | 270.372 | 1 | | 0.000 | 0.4 | 0.374 | 0.461 |
| Share - Rented housing | -0.475 | 0.054 | 78.143 | 1 | | 0.000 | 0.6 | 0.560 | 0.691 |
|  |  |  |  |  | |  |  |  |  |
| Education level | | | | | | | | | |
| Share - University educated | -0.857 | 0.144 | 35.274 | 1 | | 0.000 | 0.4 | 0.320 | 0.563 |
| Share - Upper secondary  school educated | -0.832 | 0.156 | 28.463 | 1 | | 0.000 | 0.4 | 0.321 | 0.591 |
| Share - Less than Upper  secondary school educated | -8.044 | 0.177 | 2056.047 | 1 | | 0.000 | 0.0 | 0.000 | 0.000 |
|  |  |  |  |  | |  |  |  |  |
| Income | | | | | | | | | |
| Share - Income above median | 0.445 | 0.068 | 42.190 | 1 | | 0.000 | 1.6 | 1.364 | 1.784 |
| Share - Income below median | -1.293 | 0.084 | 236.512 | 1 | | 0.000 | 0.3 | 0.233 | 0.324 |
|  |  |  |  |  | |  |  |  |  |
| Age | | | | | | | | | |
| Share - 20-29 years old | -0.042 | 0.112 | 0.141 | 1 | | 0.707 | 1.0 | 0.770 | 1.194 |
| Share - 30-39 years old | 0.785 | 0.193 | 16.496 | 1 | | 0.000 | 2.2 | 1.501 | 3.204 |
| Share - 40-64 years old | 1.658 | 0.168 | 96.970 | 1 | | 0.000 | 5.2 | 3.773 | 7.300 |
| Share - > 64 years old | 1.008 | 0.086 | 136.090 | 1 | | 0.000 | 2.7 | 2.314 | 3.247 |
|  |  |  |  |  | |  |  |  |  |
| Population density | | | | | | | | | |
| Sparsely populated |  |  | 316.411 | 2 | | 0.000 |  |  |  |
| Semi densely populated | -0.200 | 0.015 | 179.470 | 1 | | 0.000 | 0.8 | 0.795 | 0.843 |
| Densely populated | -0.183 | 0.014 | 168.972 | 1 | | 0.000 | 0.8 | 0.810 | 0.856 |
|  |  |  |  |  | |  |  |  |  |
| Other | | | | | | | | | |
| Share - Born in Sweden | -0.683 | 0.027 | 654.451 | 1 | | 0.000 | 0.5 | 0.480 | 0.532 |
| Share - Non-employed | 1.585 | 0.133 | 141.493 | 1 | | 0.000 | 4.9 | 3.759 | 6.338 |
| Share - Female | -1.655 | 0.118 | 197.199 | 1 | | 0.000 | 0.2 | 0.152 | 0.241 |
|  |  |  |  |  | |  |  |  |  |
| Constant | 2.668 | 0.097 | 753.515 | 1 | | 0.000 | 14.4 |  |  |
|  |  |  |  |  | |  |  |  |  |
| N | 1,864,372 | |  |  | |  |  |  |  |
| -2 Log likelihood | 2,302,813.149 | |  |  | |  |  |  |  |
| Cox & Snell R Square | 0.100 | |  |  | |  |  |  |  |
| Nagelkerke R Square | 0.136 | |  |  | |  |  |  |  |

Table S2. Results from model 1 for Period 3 (autumn) for Public transport travel patterns (yellow) and socioeconomic data (green).

| Period 3 | | | | | | | | |
| --- | --- | --- | --- | --- | --- | --- | --- | --- |
| Variable | **B** | **S.E.** | **Wald** | **df** | **Sig.** | **Exp(B)** | **95% C.I.for Exp(B)** | |
|  |  |  |  |  |  |  | **Lower** | **Upper** |
| Travel pattern | | | | | | | | |
| Number of journeys pre-COVID | 0.012 | 0.000 | 2308.501 | 1 | 0.000 | 1.013 | 1.012 | 1.013 |
| Number of active days pre-COVID | -0.072 | 0.001 | 8553.201 | 1 | 0.000 | 0.931 | 0.929 | 0.932 |
|  |  |  |  |  |  |  |  |  |
| Ticket type | | | | | | | | |
| 30 Days Travel card | 0.610 | 0.014 | 2027.275 | 1 | 0.000 | 1.840 | 1.791 | 1.889 |
| 90 Days Travel card | 0.578 | 0.016 | 1306.332 | 1 | 0.000 | 1.8 | 1.728 | 1.839 |
| Single ticket | 0.336 | 0.013 | 647.960 | 1 | 0.000 | 1.4 | 1.364 | 1.436 |
| Yearly Travel card | -0.321 | 0.016 | 418.092 | 1 | 0.000 | 0.7 | 0.703 | 0.748 |
| Visitor Travel card | 2.060 | 0.020 | 11050.524 | 1 | 0.000 | 7.8 | 7.550 | 8.153 |
| Youth Travel card | 0.760 | 0.014 | 2887.441 | 1 | 0.000 | 2.1 | 2.079 | 2.198 |
|  |  |  |  |  |  |  |  |  |
| Housing conditions | | | | | | | | |
| Share - Owned housing | -1.013 | 0.051 | 389.092 | 1 | 0.000 | 0.4 | 0.328 | 0.401 |
| Share - Cooperative apartment | -0.934 | 0.052 | 328.210 | 1 | 0.000 | 0.4 | 0.355 | 0.435 |
| Share - Rented housing | -0.561 | 0.052 | 116.660 | 1 | 0.000 | 0.6 | 0.516 | 0.632 |
|  |  |  |  |  |  |  |  |  |
| Education level | | | | | | | | |
| Share - University educated | -1.533 | 0.139 | 122.061 | 1 | 0.000 | 0.2 | 0.164 | 0.283 |
| Share - Upper secondary  school educated | -1.537 | 0.150 | 104.734 | 1 | 0.000 | 0.2 | 0.160 | 0.289 |
| Share - Less than Upper  secondary school educated | -5.840 | 0.171 | 1166.596 | 1 | 0.000 | 0.0 | 0.002 | 0.004 |
|  |  |  |  |  |  |  |  |  |
| Income | | | | | | | | |
| Share - Income above median | 0.349 | 0.066 | 27.659 | 1 | 0.000 | 1.4 | 1.245 | 1.614 |
| Share - Income below median | -1.144 | 0.081 | 197.431 | 1 | 0.000 | 0.3 | 0.272 | 0.374 |
|  |  |  |  |  |  |  |  |  |
| Age | | | | | | | | |
| Share - 20-29 years old | 0.486 | 0.108 | 20.167 | 1 | 0.000 | 1.6 | 1.315 | 2.009 |
| Share - 30-39 years old | 1.878 | 0.186 | 101.576 | 1 | 0.000 | 6.5 | 4.541 | 9.428 |
| Share - 40-64 years old | 2.458 | 0.162 | 229.085 | 1 | 0.000 | 11.7 | 8.498 | 16.061 |
| Share - > 64 years old | 1.166 | 0.084 | 194.452 | 1 | 0.000 | 3.2 | 2.724 | 3.780 |
|  |  |  |  |  |  |  |  |  |
| Population density | | | | | | | | |
| Sparsely populated |  |  | 214.465 | 2 | 0.000 |  |  |  |
| Semi densely populated | -0.188 | 0.014 | 169.959 | 1 | 0.000 | 0.8 | 0.805 | 0.852 |
| Densely populated | -0.109 | 0.014 | 63.280 | 1 | 0.000 | 0.9 | 0.873 | 0.921 |
|  |  |  |  |  |  |  |  |  |
| Other | | | | | | | | |
| Share - Born in Sweden | -0.665 | 0.026 | 663.223 | 1 | 0.000 | 0.5 | 0.489 | 0.541 |
| Share - Non-employed | 1.085 | 0.129 | 70.894 | 1 | 0.000 | 3.0 | 2.299 | 3.809 |
| Share - Female | -2.412 | 0.113 | 452.250 | 1 | 0.000 | 0.1 | 0.072 | 0.112 |
|  |  |  |  |  |  |  |  |  |
| Constant | 2.509 | 0.094 | 715.135 | 1 | 0.000 | 12.3 |  |  |
|  |  |  |  |  |  |  |  |  |
| N | 1,864,372 |  |  |  |  |  |  |  |
| -2 Log likelihood | 2,446,311.089 |  |  |  |  |  |  |  |
| Cox & Snell R Square | 0.039 |  |  |  |  |  |  |  |
| Nagelkerke R Square | 0.052 |  |  |  |  |  |  |  |

- 1. Model 2

Table S3. Results from model 2 for Period 2 (spring) for Public transport travel patterns (yellow) and the clusters created from the socioeconomic data (green).

| Period 2 | | | | | | | | |
| --- | --- | --- | --- | --- | --- | --- | --- | --- |
| Variable | **B** | **S.E.** | **Wald** | **df** | **Sig.** | **Exp(B)** | **95% C.I.for Exp(B)** | |
|  |  |  |  |  |  |  | **Lower** | **Upper** |
| Travel pattern | | | | | | | | |
| Number of journeys pre-COVID | -0.004 | 0.000 | 193.194 | 1 | 0.000 | 0.996 | 0.996 | 0.997 |
| Number of active days pre-COVID | -0.063 | 0.001 | 6474.444 | 1 | 0.000 | 0.939 | 0.937 | 0.940 |
|  |  |  |  |  |  |  |  |  |
| Ticket type | | | | | | | | |
| 30 Days Travel card | 0.330 | 0.014 | 582.380 | 1 | 0.000 | 1.391 | 1.354 | 1.429 |
| 90 Days Travel card | 0.328 | 0.016 | 412.915 | 1 | 0.000 | 1.388 | 1.344 | 1.432 |
| Single ticket | 0.443 | 0.013 | 1095.574 | 1 | 0.000 | 1.557 | 1.517 | 1.598 |
| Yearly Travel card | 0.003 | 0.016 | 0.032 | 1 | 0.858 | 1.003 | 0.972 | 1.034 |
| Visitor Travel card | 2.080 | 0.021 | 9840.419 | 1 | 0.000 | 8.007 | 7.685 | 8.343 |
| Youth Travel card | -0.207 | 0.014 | 210.227 | 1 | 0.000 | 0.813 | 0.791 | 0.836 |
|  |  |  |  |  |  |  |  |  |
| Clusters | | | | | | | | |
| Cluster 1 | -0.335 | 0.006 | 2798.252 | 1 | 0.000 | 0.715 | 0.706 | 0.724 |
| Cluster 2 | -0.139 | 0.005 | 801.335 | 1 | 0.000 | 0.871 | 0.862 | 0.879 |
| Cluster 3 | -0.430 | 0.011 | 1667.629 | 1 | 0.000 | 0.650 | 0.637 | 0.664 |
| Cluster 4 | 0.088 | 0.005 | 377.723 | 1 | 0.000 | 1.093 | 1.083 | 1.102 |
| Cluster 5 |  |  | 8909.748 | 4 | 0.000 |  |  |  |
|  |  |  |  |  |  |  |  |  |
| Constant | 0.693 | 0.014 | 2477.251 | 1 | 0.000 | 2.000 |  |  |
|  |  |  |  |  |  |  |  |  |
| N | 1,864,372 |  |  |  |  |  |  |  |
| -2 Log likelihood | 2,312,402.339 |  |  |  |  |  |  |  |
| Cox & Snell R Square | 0.096 |  |  |  |  |  |  |  |
| Nagelkerke R Square | 0.130 |  |  |  |  |  |  |  |

Table S4. Results from model 2 for Period 3 (autumn) for Public transport travel patterns (yellow) and the clusters created from the socioeconomic data (green).

| Period 3 | | | | | | | | |
| --- | --- | --- | --- | --- | --- | --- | --- | --- |
| Variable | **B** | **S.E.** | **Wald** | **df** | **Sig.** | **Exp(B)** | **95% C.I.for Exp(B)** | |
|  |  |  |  |  |  |  | **Lower** | **Upper** |
| Travel pattern | | | | | | | | |
| Number of journeys pre-COVID | 0.013 | 0.000 | 2413.981 | 1 | 0.000 | 1.013 | 1.012 | 1.013 |
| Number of active days pre-COVID | -0.074 | 0.001 | 9147.674 | 1 | 0.000 | 0.929 | 0.927 | 0.930 |
|  |  |  |  |  |  |  |  |  |
| Ticket type | | | | | | | | |
| 30 Days Travel card | 0.551 | 0.013 | 1671.628 | 1 | 0.000 | 1.735 | 1.690 | 1.781 |
| 90 Days Travel card | 0.523 | 0.016 | 1078.896 | 1 | 0.000 | 1.688 | 1.636 | 1.741 |
| Single ticket | 0.297 | 0.013 | 508.975 | 1 | 0.000 | 1.346 | 1.311 | 1.381 |
| Yearly Travel card | -0.374 | 0.016 | 572.417 | 1 | 0.000 | 0.688 | 0.667 | 0.709 |
| Visitor Travel card | 2.045 | 0.020 | 10956.414 | 1 | 0.000 | 7.726 | 7.436 | 8.027 |
| Youth Travel card | 0.702 | 0.014 | 2485.712 | 1 | 0.000 | 2.017 | 1.962 | 2.074 |
|  |  |  |  |  |  |  |  |  |
| Clusters | | | | | | | | |
| Cluster 1 | -0.008 | 0.006 | 1.766 | 1 | 0.184 | 0.992 | 0.980 | 1.004 |
| Cluster 2 | -0.035 | 0.005 | 53.509 | 1 | 0.000 | 0.966 | 0.957 | 0.975 |
| Cluster 3 | -0.263 | 0.010 | 665.964 | 1 | 0.000 | 0.769 | 0.754 | 0.784 |
| Cluster 4 | 0.136 | 0.004 | 967.103 | 1 | 0.000 | 1.145 | 1.136 | 1.155 |
| Cluster 5 |  |  | 3549.374 | 4 | 0.000 |  |  |  |
|  |  |  |  |  |  |  |  |  |
| Constant | 0.265 | 0.014 | 374.175 | 1 | 0.000 | 1.303 |  |  |
|  |  |  |  |  |  |  |  |  |
| N | 1,864,372 |  |  |  |  |  |  |  |
| -2 Log likelihood | 2,455,803.153 |  |  |  |  |  |  |  |
| Cox & Snell R Square | 0.034 |  |  |  |  |  |  |  |
| Nagelkerke R Square | 0,045 |  |  |  |  |  |  |  |
